# Using Milk Urea Nitrogen to Evaluate Diet Formulation and Environmental Impact on Dairy Farms

**DOI:** 10.1100/tsw.2001.265

**Published:** 2001-10-18

**Authors:** J.S. Jonker, R.A. Kohn

**Affiliations:** United States Environmental Protection Agency, Washington, DC 20460, USA

**Keywords:** nitrogen, dairy cattle, milk urea nitrogen, nonpoint source

## Abstract

Reducing nitrogen (N) excretion by dairy cattle is the most effective means to reduce N losses (runoff, volatilization, and leaching) from dairy farms. The objectives of this review are to examine the use of milk urea nitrogen (MUN) to measure N excretion and utilization efficiency in lactating dairy cows and to examine impacts of overfeeding N to dairy cows in the Chesapeake Bay drainage basin. A mathematical model was developed and evaluated with an independent literature data set to integrate MUN and milk composition to predict urinary and fecal excretion, intake, and utilization efficiency for N in lactating dairy cows. This model was subsequently used to develop target MUN concentrations for lactating dairy cattle fed according to National Research Council (NRC) recommendations. Target values calculated in this manner were 8 to 14 mg/dl for a typical lactation and were most sensitive to change in milk production and crude protein intake. Routine use of MUN to monitor dairy cattle diets was introduced to dairy farms (n = 1156) in the Chesapeake Bay watershed. Participating farmers (n = 454) were provided with the results of their MUN analyses and interpretive information monthly for a period of 6 months. The average MUN across all farms in the study increased in the spring, but the increase was 0.52 mg/dl lower for farmers receiving MUN results compared to those who did not participate in the program. This change indicated that participating farmers reduced N feeding compared to nonparticipants. Average efficiency of feed N utilization (N in milk / N in feed x 100) was 24.5% (SD = 4.5). On average, farmers fed 6.6% more N than recommended by the NRC, resulting in a 16% increase in urinary N and a 2.7% increase in fecal N compared to feeding to requirement. N loading to the Chesapeake Bay from overfeeding protein to lactating dairy cattle was estimated to be 7.6 million kg/year. MUN is a useful tool to measure diet adequacy and environmental impact from dairy farms.

## INTRODUCTION

Due to significant stress from pollution in the Chesapeake Bay, in 1976 Congress directed the U.S. Environmental Protection Agency (USEPA) to undertake a comprehensive study of the Bay's resources and water quality, and to identify appropriate measures to improve the quality of the Bay[1]. This initiated the establishment of the Chesapeake Bay Program with participation from Virginia, Delaware, Maryland, Pennsylvania, the District of Columbia, and the Federal government. In 1983, the Chesapeake

Bay Program identified eutrophication as the primary water quality issue in the Chesapeake Bay[1]. The Chesapeake Bay Program set a goal of reducing controllable sources of nitrogen (N) and phosphorus (P) entering the Bay by 40%.

Nonpoint nutrient loading to surface water is a major environmental problem in the Chesapeake Bay drainage basin[2]. Dairy farming is a large enterprise in the region, making dairy cows (n = 758,347)[3] a major contributor to nonpoint N loading[4]. Much manure and fertilizer nutrients applied to crops run off, leach, or volatilize, contributing to nutrient loading even when nutrients are optimally managed. Kohn et al.[5] determined, with a simple mathematical model, that improving animal N utilization efficiency by 50% through better herd management would reduce N losses to water by 40%. Improving manure N utilization efficiency by 100% would reduce N losses to water by only 14%[5]; however, to completely minimize nutrient impacts, feeding, crop, and manure management should be addressed in any nutrient management plan. The effects of several management practices on nutrient utilization efficiency and excretion in research dairy herds have previously been studied, such as animal grouping[6], bovine somatotropin, milking three times daily, or extended photoperiod[7]. Continuing development of new technologies that improve N utilization of dairy cows will be beneficial to decrease N loading to Chesapeake Bay.

The objectives of this review are to examine the use of milk urea nitrogen (MUN) as a means to estimate N excretion and utilization efficiency for lactating dairy cows and to examine impacts of overfeeding N to dairy cows in the Chesapeake Bay watershed.

## MILK UREA NITROGEN

Absorbed N in the blood stream of a dairy cow results from the diffusion of ammonia across the rumen wall and transport of amino acids and peptides from the small intestine[8]. Ammonia is toxic to the cow and is rapidly converted to urea in the liver[9]. Absorbed amino acids and peptides that are not utilized for maintenance, milk synthesis, growth, or fetal development are deaminated in the liver for energy, and the N converted to urea[9]. This urea becomes part of the blood urea N pool.

The blood urea N pool has three ultimate fates: recycling, secretion in milk, or excretion in urine. Recycling of urea via saliva, mucins of saliva, and across the rumen wall can be an important source of N for microbial protein synthesis in ruminants[8]. Some recycled urea diffuses into the hindgut where it is hydrolyzed and may be reabsorbed or incorporated into microbial cells excreted in the feces. Urea is filtered from the blood by the kidney and is excreted from the body in urine[10]. Blood flow through the kidney is constant within an animal[9], which ensures a constant blood filtration rate (milliliters of blood filtered per minute) regardless of urine volume.

Because urea is a small neutral molecule, it readily diffuses across cellular membranes. As milk is formed in the mammary gland, urea diffuses into and out of the mammary gland, equilibrating with urea in the blood. Because of this process, MUN equilibrates with and is proportional to blood urea N[11,12,13]. Because urea excretion is proportional to blood urea N concentration[14], total urinary N excretion has been shown to have a positive linear relationship with MUN[15,16,17].

Whereas N from excess dietary protein is excreted in urine, MUN may be used as a management tool to monitor nutritional status of lactating dairy cows and improve dairy herd nutrition. Several researchers have explored the relationship of MUN to dietary protein and energy. Variation in MUN has been suggested to be related to the protein-to-energy ratio of the diet consumed[12,18,19]. The concentration of MUN was only slightly affected by N intake when the protein-to-energy ratio was held constant, but increased with an increase in this ratio[12,19]. More recent research[11] analyzing data from 35 conventional lactation trials showed no effect of total energy (Mcal/day and nonprotein N intake (g/day) or dietary concentration of energy (Mcal/kg) and neutral detergent fiber (%) in single-factor regression analysis. The protein-to-energy ratio was significant in the study[11].

With adequate energy in the diet, MUN is indicative of protein status[13]. Roseler et al.[13] observed an increase in MUN concentration for dairy cows when different forms of protein were fed in excess of National Research Council (NRC) recommendations[20] with no difference in milk production. Conversely when protein was fed below recommendations, MUN concentration and milk production were reduced[13] because N was limiting in the diet. High levels of readily degraded protein was reported to increase MUN concentrations[21]. In a 17-farm study, Garcia et al.[22] reported MUN was better correlated to soluble intake or crude protein percentage in the diet than with the total amount of either fraction consumed. Properly balancing protein in diets reduces MUN variation and can increase milk production.

## PREDICTING URINARY AND FECAL N, INTAKE, AND UTILIZATION EFFICIENCY

Jonker et al.[16] developed and evaluated a model to estimate urinary and fecal N excretion, N intake, and N utilization efficiency for lactating dairy cows. The model requires knowledge of milk production per cow, milk protein percentage, and MUN. Urinary N is predicted as a function of MUN. Originally, urinary N (g/day) was predicted as $12.54 \times$ MUN (mg/dl) for typical Holstein cows[16]. These researchers recognized that urinary N was underpredicted for smaller breeds when using the model, but were unable to account for these effects using the data available. In September 1998, Dairy Herd Improvement Association laboratories changed the way standards were derived in the U.S. As a result, reported MUN values decreased by an average of 4 mg/dl[23]. Kauffman and St-Pierre[24] were able to account for body weight effects and the change in MUN analysis. Currently, urinary N (g/day) can best be predicted as $0.026 \times$ body weight (kg) $\times$ MUN for any breed of dairy cow.

Jonker et al.[16] also showed that the proportion of N absorbed in the body, as opposed to excreted in feces, is consistent across various types of feedstuffs. Therefore, assuming that most N is either secreted in milk or urine by mature dairy cows, N intake (g/day) can be predicted as (urinary N [g/day] + milk N + 97) / 0.83. The endogenous losses are represented as 97 g/day and the fraction of feed N digested is assumed to be 0.83. Since all intake N in mature (not growing) cows must eventually leave the animal, fecal N can be predicted as intake N – urinary N – milk N. Finally, N utilization efficiency (%) for mature cows is equal to milk N × 100 / N intake. This model was evaluated using data from several published research studies[16].

## TARGET MUN CONCENTRATIONS

Target MUN concentrations were determined for cows fed according to NRC[20] recommendations. Required N intake was calculated throughout a standard 305-day lactation for cows fed diets balanced for different forms of protein according to the NRC[20]. Driving variables used to calculate N intake requirements were milk production (kg/day), milk fat (%), body weight (kg), live weight change (kg/day), parity (1, 2, or 3+), and days pregnant. Typical lactation curves for daily milk production, milk fat percentage, milk protein percentage, and body weight change were developed from Wood's Equation[25,26] using data from the Lancaster Dairy Herd Improvement Association (702 herds, 47,378 cows, 133,057 observations) collected between July 1996 and April (1998)

Target MUN concentrations were determined for a 600-kg second lactation cow. The protein required was assumed to be that needed by the 83rd percentile cow with respect to protein requirements for the entire milking herd according to lead factors developed by Stallings and McGilliard[27]. The data represented in the current paper are adjusted for the modification in the procedure recommended by Kauffman and St-Pierre[24] and Kohn et al.[23]. A 10,000-kg lactation was assumed with 3.5% milk fat and 3.0% milk protein. The lactation curve for target MUN concentration (Fig. 1) resembles a typical lactation curve for milk production. Target MUN concentration begins low in early lactation (not 0) and increases rapidly to peak in early lactation, mirroring the rapid increase in milk production because of increasing protein intake[28]. Mean MUN concentration predicted by the model weighted by milk production for the entire 10,000-kg lactation was 10.6 mg/dl, as modified by Kauffman and St-Pierre[24,28]. Peak MUN concentration of 11.6 mg/dl occurred at 78 days in milk (DIM). In contrast, peak milk production occurred at 65 DIM. Differences between DIM for peak milk production and peak MUN concentration is due to the short time lag between peak milk production and peak dry matter (and therefore protein) intake[20].

With higher average milk production, target MUN levels increased (Fig. 1). Mean MUN weighted by milk production for a 12,000-kg lactation was 12.7 mg/dl with a peak MUN concentration of 14.5 mg/dl occurring on day 76[28]. Milk production drives the requirement for N in lactating dairy cows fed according to the NRC[20]. As milk production increases, when cows are fed according to NRC recommendations, predicted MUN concentrations increase linearly because of higher N intake and N excretion. Consequently, target MUN concentrations are extremely sensitive to changes in milk production.

Target MUN concentrations were much less sensitive to changes in milk fat and protein percentages, body weight, and parity[28]. Rodriguez et al.[29] reported lower MUN content in milk from Jersey cows compared with the MUN content of milk from Holstein cows, and Ferguson et al.[30] observed Jersey cows to have higher MUN than Holstein cows from Pennsylvania Dairy Herd Improvement Association data. These discrepancies were likely due to four factors: body weight, milk production, milk fat and protein percentage, and N intake. Renal clearance rates and blood volume may increase as animal size increases[9] and could affect differences observed between breeds as well. These target values are limited to lactating cows weighing 600 kg. Integrating effect of body weight on protein requirements[28] with effect of body weight on the relationship between MUN and urinary N excretion[24] enables calculation of the target MUN for smaller or larger cattle. A Jersey cow with a body weight of 400 kg would be expected to have a mean MUN that is 3 mg/dl higher than a Holstein with a body weight of 600 kg for the same production level.

Protein feeding level with regard to NRC[20] protein requirements affects target MUN concentrations the most. Feeding above NRC recommendations for N intake by 10% results in an increase in lactational MUN concentration of 26%[28]. This excess N intake results in elevated feed costs and excess urinary N excreted to the environment. This response clearly demonstrates that MUN is very sensitive to overfeeding protein and will be useful in field applications.

While this method provides a precise number for target MUN concentrations, an acceptable range around the target exists[28]. For a 25-cow management group, a group's MUN concentration could be ±2 mg/dl from the target concentration and still be considered within the target range (Fig. 1). Overall under typical production conditions, most dairy farms should have MUN concentrations from 8 to 12 mg/dl.

## MUN PILOT PROJECT

A confidential mail survey[31] was conducted in December 1998 with members of the Maryland and Virginia Milk Producers Cooperative (West Reston, VA; n = 1156). Participants returning the survey were offered monthly bulk tank milk analysis of MUN for 6 months as an incentive. Bulk tank MUN analyses were performed monthly for 6 months for all dairy farms from December 1998 through May 1999, regardless of survey participation. Dairy farms that completed the survey were provided their MUN concentration and interpretive information monthly.

The mean and standard deviation in N feeding parameters were calculated based on model predictions from the survey data and December milk analysis. N intake, urinary and fecal N, and N utilization efficiency were determined for each herd using the model of Jonker et al.[16] except prediction of urinary N was equal to 0.0259 × body weight × MUN as recommended by Kauffman and St-Pierre[32]. Crude protein requirements were determined using NRC[20] recommendations for dairy cattle assuming a one-group total mixed ration (TMR) was fed[28]. The protein required was assumed to be that needed by the 83rd percentile cow with respect to protein requirements for the entire milking herd[27]. This approach prevents underfeeding of most cows. Excess N feeding was determined as the difference between observed N intake and that predicted to be required.

A total of 472 dairy farmers responded to the survey for a 40.8% rate of return. More than 60% of the responding dairy farms indicated prior knowledge of MUN. Prior to the survey, however, more than 89.5% of the dairy farms did not routinely test for MUN. A total of 33 dairy nutrition consultants responded to the survey for a 50% return rate. Conversely to the lack of use among dairy farmers, 88% of consultants recommended routine use of MUN.

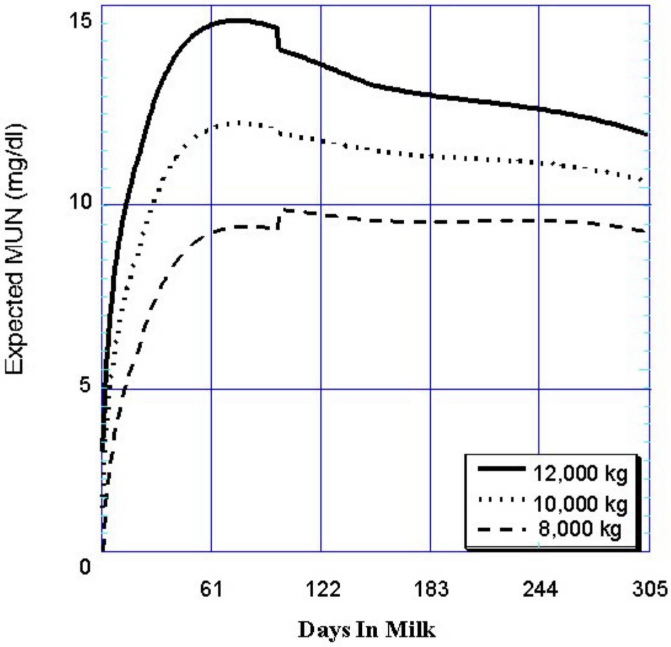

**FIGURE 1.** Predicted MUN (mg/dl) throughout a 305-day lactation for milk production of 12,000 kg (———), 10,000 kg/year (·······) and 8,000 kg/year (- - - -).

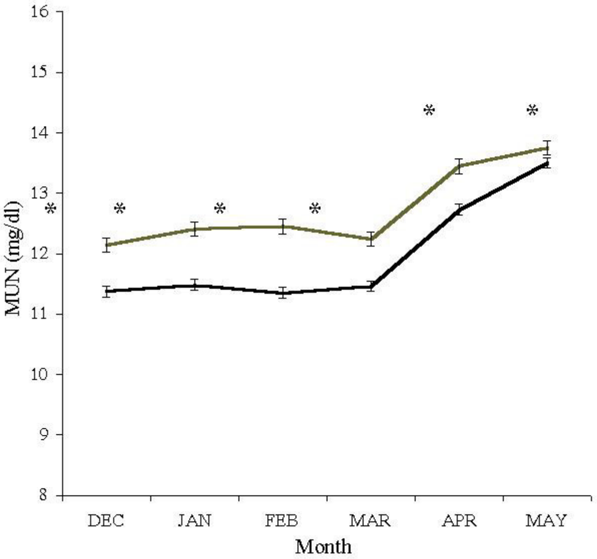

**FIGURE 2.** Monthly MUN (mg/dl) concentrations for dairy farms that participated in the program (top line) compared to those who choose not to participate (bottom line). *Contrasts by month differ by $p < 0.05$.

Observed MUN was 12.7 mg/dl, but feeding according to the NRC[20] and allowing for variation within the herd by feeding the 83rd percentile cow would have resulted in a MUN of 11.0 mg/dl. Farmers appeared to feed 6.6% more N than recommended by the NRC[20]. This overfeeding resulted in a 16% increase in urinary N and a 2.7% increase in fecal N compared to feeding to requirements. Most farmers (71.5%) appeared to feed more than recommended amounts of protein by an average of 61 g/day or 11% of required N. Urinary N excretion ranged from 143 g/day for the 17th percentile herd to 247 g/day for the 83rd percentile herd. Similarly, herd efficiency ranged between the same percentiles from 24.5 to 32.3%. The tendency to overfeed and herd N efficiency were not associated with herd size ($p > 0.1$).

Participants in the program initially had higher MUN values than nonparticipants, perhaps reflecting higher-producing herds among participants (Fig. 2). For both groups of farms, MUN increased in the spring when lush pastures high in protein were available and when milk production is higher. However, MUN did not increase as much among participants in

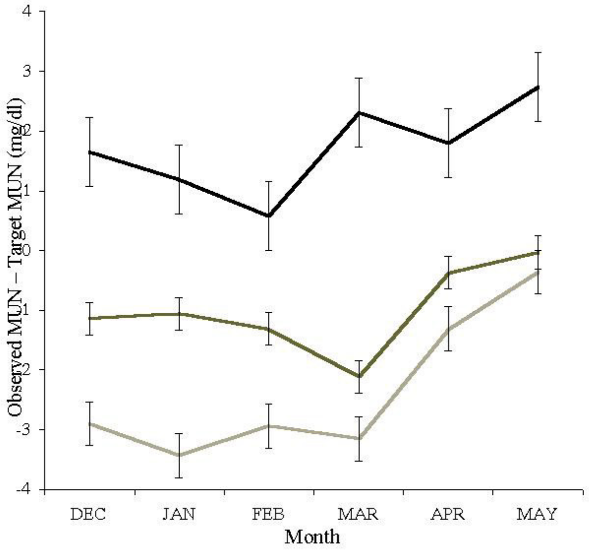

**FIGURE 3.** Monthly MUN minus target MUN for dairy farms that reported decreasing dietary crude protein (top line), increasing dietary crude protein (bottom line), or not changing dietary crude protein (middle line).

the study as for nonparticipants (Fig. 2). Thus, it appears the study encouraged some farmers to reduce protein feeding levels.

As was hoped, farmers that appeared to be underfeeding protein appeared to increase protein feeding during the course of the program, and farmers that appeared to be overfeeding protein appeared to decrease diet protein relative to nonparticipants (Fig. 3). For farms that indicated they increased dietary crude protein (% DM), MUN was lower compared to target during the first 3 months of the program (Fig. 3) and MUN increased during the last 3 months, suggesting an increase in dietary crude protein. For dairy farmers that indicated they decreased dietary crude protein (% DM), MUN was higher than target values and MUN appeared to increase in the spring, but the magnitude of the spring increase was 1 mg/dl lower than for nonparticipating farms.

## ECONOMIC AND ENVIRONMENTAL IMPACT OF OVERFEEDING PROTEIN

The environmental and economic impact of overfeeding dairy herds in the Chesapeake Bay drainage basin were estimated on summarized results from December 1998 MUN analyses according to the method of Jonker and Kohn[33]. Excess N fed in the watershed was calculated by multiplying the number of cows in the watershed during the study (n = 758,347)[3] by the fraction of farms overfeeding N and the average excess N overfed per cow. The cost or savings of feed N was estimated by assuming that the change in crude protein intake was orchestrated by substituting corn grain with soybean meal (44%) or vise versa. The 5-year average prices (1996 to 2000) for soybean meal ($0.210/kg) and corn grain ($0.097/kg) were used[34]. On a dairy farm, 75% of manure N was expected to be lost from runoff, volatilization, leaching, and denitrification[5]. In addition, losses are reduced by being able to grow less crop protein, but these were not considered in the present study because the excess protein was likely to be soybean meal that was imported into the region

The average cow involved in the pilot project produced 28 kg of milk per day, and excreted 187 g/day fecal N, and 195 g/day urinary N[35]. Multiplying this by the number of cows in the watershed during the study (n = 758,347)[3] provides an estimate of the total N excreted by dairy cows as 105 million kg N/year. This estimate does not include manure production by calves and prelactation heifers. Because only 25% of manure N is effectively recycled back to the herd in harvested crops, as much as 75% of this manure N may be lost to the environment. Additional N may be lost to the environment during the production of feeds for these cattle. Some N may enter groundwater or the atmosphere or be denitrified to $N_2$ gas rather than contributing to surface water eutrophication. Nonetheless, dairy farms are likely to contribute a substantial amount of N to Chesapeake Bay. The total nonpoint source N entering Chesapeake Bay was estimated to be 96 million kg/year[2]. If only 50% of dairy manure N eventually entered Bay waters through runoff, volatilization, and precipitation, or leaching to groundwater and resurfacing, more than half the estimated total nonpoint source N would come from dairy manure. Considering all the potential sources of nonpoint N (e.g., other animal and crop agriculture, septic systems), estimates of total nonpoint source N may be too low.

Estimates of the environmental and economic impact of overfeeding N in the watershed are presented in Table 1, which shows that 71.5% of farms fed N above NRC[20] recommendations for the 83$^{rd}$ percentile cow. This excess N would be excreted in urine. Since less than 25% of excreted N is typically available to be recycled to crops, 75% of the manure N is likely to be lost to the environment. Thus, 7.6 million kg of N would have been lost to water resources due to overfeeding of N by farmers. This figure represents 7.9% of the total nonpoint source N loaded to Chesapeake Bay each year[2]. In addition, crops would be grown to produce this excess feed N, and N losses would result from the fields where these crops were produced. The cost of feeding excess soybean meal in place of corn grain was $32.94 per cow per year, or $17.86 million/year. Theoretically some of that cost could be recovered with increased milk production from the highest producing cows[6].

Clearly MUN can be used both as a nutritional tool by dairy farmers to identify when cows are consuming excess protein and to quantify nonpoint source N emanating from dairy farms. A potential exists to both increase dairy farm profitability and decrease nonpoint N loading to the environment. However, many dairy farms maintain high production with lower MUN concentrations than the target, indicating a potential for feeding below NRC recommendations and further reducing N loading.

## USING MUN FOR DIET EVALUATION

High MUN levels are often attributed to specific causes, including too much RDP, too little energy, and imbalance of carbohydrate and protein ratios, not enough RUP. None of these reasons alone tells the complete story; high (or low) MUN concentrations depend on a combination of factors. In simplest terms, high MUN concentrations indicate a general excess N intake for a given level of milk production[16]. Excess N might be the result of excess protein[11,13,16,22,36]. The wasted protein is excreted in the cow's urine[16] resulting in lost income to the dairy farmer[33]. With an imbalance of available protein to fermentable carbohydrate[11,12,19,22], energy may be limiting in the diet and the cow's milk production reduced. Because of this reduced production, the protein cannot be used, and high MUN results.

Under typical production conditions, most dairy herds should have MUN concentrations from 8 to 12 mg/dl. When the average MUN concentration is outside the target range, the cause needs to be determined. A minimum of 25 cows should be sampled from a management group to determine an average MUN value for that group. Bulk tank samples may save money, but will not show differences among different management groups of cows.

The first area to consider when MUN concentrations are outside the target range is milk production (Table 2). Are the

**TABLE 1**
**Economic and Environmental Impact of Overfeeding Protein to Dairy Cows in the Chesapeake Bay Drainage Basin**

| Item | Estimate |
|---|---|
| Farms feeding N above recommendations[1] | 71.5% |
| Excess N per overfed cow[1], kg/year | 18.6 |
| Excess N fed in watershed, $10^6$ kg/year | 10.1 |
| N loss to Bay from overfeeding[2], $10^6$ kg/year | 7.6 |
| Additional feed cost per overfed cow[3], $/year | $32.94 |
| Cost of overfeeding in Watershed, $10^6$ $/year | $17.86 |

[1] N intake – N recommended.
[2] N losses from manure application and crop production minus estimated denitrification.
[3] Cost of excess soybean meal to exceed crude protein requirement.

**TABLE 2**
**Check List to Identify Causes of High or Low MUN Concentrations**

| | |
|---|---|
| 1. Milk production | Are the cows producing as much milk as expected? |
| 2. Ration formulation | Is the ration formulated to meet the cows' nutrient requirements? |
| 3. Feed analysis | Are all forages analyzed routinely? |
| 4. Feed digestibility | Do any of the feeds have heat damage? |
| 5. Feeding management | Are the cows fed the diet as formulated or is something lost in the translation from nutritionist to manager to feeder? |
| 6. Animal consumption | Are the cows eating what is offered or are they selecting part of the ration? |

cows producing what they are expected to produce and what the ration is balanced for? If the cows are producing less than expected, excess protein consumption results in elevated MUN levels. The reason for lower milk production needs to be examined. Lower than expected milk production can be caused by management (e.g., too-high expectation) or ration formulation (e.g., not enough energy).

A next logical step, if milk production is as expected, is to examine the ration formulation. Is the ration formulated to meet the nutrient requirements of the cow? While computer programs have made ration formulation easier, the results are only as good as the expertise of the person performing the formulation and the accuracy of the program used. If, for example, a ration is balanced for crude protein level only and not protein fractions, a situation could arise where degradable protein level is too high, causing elevated MUN levels.

When ration formulation appears correct, differences may exist in nutrient composition of actual feed ingredients and nutrient composition used in ration balancing. Are the forages analyzed routinely and are the samples representative of the forage being fed? Nutrient composition of forages can change dramatically from field to field and cutting to cutting, so occasional forage testing may not show the true variability of the forage nutrient composition.

When accounting for these factors, high MUN concentrations may still not be explained. The actual process of feeding the cows may need to be examined. Is the TMR mixed thoroughly? An improperly mixed TMR can result in inadequate distribution of nutrients with some cows getting more than their share. Is the ration being fed according to how it was balanced? Careful attention must be made in order not to over- or underfeed any particular diet ingredient. If, for example, soybean meal is overfed and cornmeal underfed, there will be an excess of protein in the diet relative to available energy and high MUN will result.

If the cause of high MUN level is still not isolated, diet consumption by the cow needs to be examined. Are the cows consuming what they are being fed? There are really two rations to consider. The first is the ration as it is fed to the animal (assuming it is already properly balanced and mixed). The second is what the cow actually consumes. The feed left in the bunk by the cows should look like the ration that was fed to the cows earlier. If the cows are able to sort through the ration, concentrate may be consumed preferentially over forage and high MUN levels may occur.

Although low MUN levels can indicate efficient protein feeding, conditions can exist in which extremely low MUN levels may indicate a protein deficiency in the diet and potentially lost milk production. Low MUN levels (below 8 mg/dl) may suggest the cows' diet does not contain adequate available protein. Do any of the feed ingredients have heat damage reducing its digestibility? If a dried brewers grain (or other dried by-product feed) being fed is dark brown, it may have a significant portion of bound protein that the animal is unable to use. If forages were heat damaged during the ensiling or hay preservation process, the protein digestibility may be reduced. This may cause the diet to be low in absorbed protein and may result in a low MUN level. When MUN levels are extremely low, production may be limited by a protein-deficient diet. Suspicious feeds should be analyzed for acid detergent insoluble N or bound protein.

## CONCLUSIONS

MUN is an indicator of diet adequacy and N utilization efficiency in lactating dairy cattle. As a management tool for dairy farmers, MUN offers a simple and noninvasive approach to examine protein status of rations fed to dairy cattle. As demonstrated in the pilot project, through routine monitoring of MUN dairy farmers can adjust dietary protein levels to better match their cows' protein requirements and potentially increase profitability by reducing feed costs.

MUN also is an effective means to estimate N excretion from lactating dairy cattle. As demonstrated in a pilot project in the Chesapeake Bay drainage basin, MUN can be utilized to assess the impacts of excess N feeding to dairy cows in a watershed. By monitoring the routine use of MUN by dairy farmers, changing N impacts of dairy farms on a watershed can be determined.

| | | |
|---|---|---|
| Received: | July | 23, 2001 |
| Revised: | September | 18, 2001 |
| Accepted: | October | 3, 2001 |
| Published: | October | 18, 2001 |
